# The More the Better? A Comparison of the Information Sources Used by the Public during Two Infectious Disease Outbreaks

**DOI:** 10.1371/journal.pone.0140028

**Published:** 2015-10-20

**Authors:** Cynthia G. Jardine, Franziska U. Boerner, Amanda D. Boyd, S. Michelle Driedger

**Affiliations:** 1 School of Public Health, University of Alberta, Edmonton, Alberta, Canada; 2 Institute for Technology Assessment and Systems Analysis, Karlsruhe Institute of Technology, Karlsruhe, Germany; 3 The Edward R. Murrow College of Communication, Washington State University, Pullman, Washington, United States of America; 4 Community Health Sciences, University of Manitoba, Winnipeg, Manitoba, Canada; University of Hong Kong, HONG KONG

## Abstract

Recent infectious disease outbreaks have resulted in renewed recognition of the importance of risk communication planning and execution to public health control strategies. Key to these efforts is public access to information that is understandable, reliable and meets their needs for informed decision-making on protective health behaviours. Learning from the trends in sources used in previous outbreaks will enable improvements in information access in future outbreaks. Two separate random-digit dialled telephone surveys were conducted in Alberta, Canada, to explore information sources used by the public, together with their perceived usefulness and credibility, during the 2003 Severe Acute Respiratory Syndrome (SARS) epidemic (n = 1209) and 2009–2010 H1N1 pandemic (n = 1206). Traditional mass media were the most used information sources in both surveys. Although use of the Internet increased from 25% during SARS to 56% during H1N1, overall use of social media was not as high as anticipated. Friends and relatives were commonly used as an information source, but were not deemed very useful or credible. Conversely, doctors and health professionals were considered credible, but not consulted as frequently. The use of five or more information sources increased by almost 60% between the SARS and H1N1 surveys. There was a shift to older, more educated and more affluent respondents between the surveys, most likely caused by a decrease in the use of landlines amongst younger Canadians. It was concluded that people are increasingly using multiple sources of health risk information, presumably in a complementary manner. Subsequently, although using online media is important, this should be used to augment rather than replace more traditional information channels. Efforts should be made to improve knowledge transfer to health care professionals and doctors and provide them with opportunities to be more accessible as information sources. Finally, the future use of telephone surveys needs to account for the changing demographics of the respondents accessed through such surveys.

## Introduction

Infectious disease outbreaks have plagued mankind for centuries, with many (such as ‘The Black Death’ and the 1918 ‘Spanish Flu’) resulting in millions of deaths world-wide. Although there have been tremendous strides in the control of communicable diseases (such as antibiotics and vaccines) in modern times, other changes (such as unprecedented population growth and increased mobility through air travel) have concurrently increased the potential for disease development and epidemic or even pandemic spread [1]. There have been several high profile international outbreaks in recent years, such as the Severe Acute Respiratory Syndrome (SARS) epidemic in 2003, the H1N1 pandemic in 2009–2010 and the recent Ebola virus disease (formerly known as Ebola haemorrhagic fever) epidemic. While Ebola was primarily confined to the most severely affected west African countries of Guinea, Sierra Leone and Liberia, an October 2014 fatal case in the United States (in a Liberian immigrant), with transmission of the disease to an American health care worker, heightened fears that the disease may spread to North America. Polls conducted in both Canada (n = 1009) [2] and the United States (n = 1577) [3] subsequent to the October 2014 case indicated that 79% and 77% of respondents, respectively, were somewhat or very concerned about Ebola transmission.

These recent outbreaks have resulted in a renewed appreciation of the need for public health agencies to both plan and act appropriately to avoid potential widespread harm from infectious diseases. Effective risk communication has been identified as key to these efforts [4] and indeed has been listed by the World Health Organization as one of the eight core capacities of the International Health Regulations (2005) [5]. However, risk communication during infectious disease outbreaks throughout the world continues to be found wanting and in need of improvement [6, 7]. For example, health care workers in both Canada [8] and the United States [9] have questioned the preparedness of public health agencies to deal with potential Ebola cases. Their concern has been attributed to a lack of communication about existing infectious disease plans and mitigation strategies [10].

Successful public health risk communication during an infectious disease outbreak is contingent upon people being able to access information that is understandable, reliable and meets their needs for informed decision-making on protective health behaviours [11]. Understanding the sources of information people use during an epidemic or pandemic, together with the perceived usefulness and credibility of that information, is important to effectively target risk communication channels and messages [12, 13].

Learning from past outbreaks, and trends between outbreaks, provides an invaluable opportunity to improve performance and mitigate impacts of future outbreaks. In this article, we report on public information use, together with assessed usefulness and credibility, in the province of Alberta, Canada during both the SARS epidemic and H1N1 pandemic. The lessons learned from these outbreaks will enable public health agencies to effect further improvement in risk communication efforts in subsequent infectious disease events.

## Background

### SARS and H1N1 Outbreaks in Canada and Alberta

The SARS outbreak began in Guangdong province, China in 2002. A doctor treating these initial patients later travelled to Hong Kong and infected several people at the hotel where he was staying, including a 78-year old woman from Canada who was in Hong Kong on holiday. Her transmission of the disease to family members upon her return to Canada led to the Toronto SARS outbreak in 2003 [14]. This outbreak occurred in two waves (March to April and April to July). Ultimately, approximately 400 people were diagnosed with the disease, 25,000 Toronto residents were placed in quarantine, and 44 people died in Canada (all in Toronto). Approximately 85% of the Canadian SARS cases occurred in Ontario, although suspect cases were also reported in the provinces of British Columbia, Alberta, New Brunswick, Prince Edward Island and Saskatchewan (Table 1) [15, 16, 17]. Intense media coverage of the Toronto SARS outbreak (as the only city affected outside of Asia) eventually led to a World Health Organization travel advisory. Partly in response to identified areas for improvement in Canada’s responsiveness to future health care crisis (including communication), in 2004 the Canadian government established the Public Health Agency of Canada, and provided increased funding for public health and pandemic influenza preparedness efforts. [16]

**Table 1:** Canadian SARS cases by province and territory (PHAC 2003).

Canadian Province/Territory	SARS Cases	
	Suspect Cases	Probable Cases
British Columbia	46	4
Alberta	6	0
Saskatchewan	1	0
Manitoba	0	0
Ontario	134	241
Quebec	0	0
Nova Scotia	0	0
Newfoundland	0	0
New Brunswick	2	0
Prince Edward Island	4	0
Northwest Territories	0	0
Yukon Territory	0	0
Nunavut	0	0
**TOTAL (Canada)**	**193**	**245**

Case Definitions (WHO 2003)

Suspect case

1. A person presenting after 1 November 2002 with history of high fever (>38°C) AND cough or breathing difficulty AND one or more of the following exposures during the 10 days prior to onset of symptoms

a. close contact with a person who is a suspect or probable case of SARS

b. history of travel, to an area with recent local transmission of SARS

c. residing in an area with recent local transmission of SARS

2. A person with an unexplained acute respiratory illness resulting in death after 1 November 2002, but on whom no autopsy has been performed AND one or more of the following exposures during to 10 days prior to onset of symptoms

a. close contact with a person who is a suspect or probable case of SARS

b. history of travel, to an area with recent local transmission of SARS

c. residing in an area with recent local transmission of SARS

Probable case

1. A suspect case with radiographic evidence of infiltrates consistent with pneumonia or respiratory distress syndrome (RDS) on chest X-ray (CXR).

2. A suspect case of SARS that is positive for SARS coronavirus by one or more assays.

3. A suspect case with autopsy findings consistent with the pathology of RDS without an identifiable cause.

In contrast, the H1N1 outbreak was more universally widespread with greater mortality across Canada. The H1N1 outbreak, originally called ‘swine flu’, is believed to have begun in Mexico in March 2009, and was first reported in Canada on April 26, 2009 [18]. On June 11, 2009, the World Health Organization (WHO) declared the outbreak to be a Phase 6 global influenza pandemic (the first since 1969). A Phase 6 pandemic, the highest level, indicates that the same identified virus has caused sustained outbreaks in two or more countries in one WHO region and in at least one other country in another WHO region [19]. By June 2010, H1N1 had been reported in 214 countries, with more than 18,000 deaths world-wide [20]. The Canadian H1N1 pandemic also occurred in two waves: the first wave peaked in June 2009 and the second in October 2009. In Canada, there were 428 deaths attributed to H1N1 [21] and more than 40,000 laboratory-confirmed cases [18]. Alberta recorded 71 deaths from H1N1 and 1,276 hospitalizations [22]. The WHO declared H1N1 to be in the post-pandemic phase on August 20, 2010.

### Communications during SARS and H1N1

Communication to the public was identified as particularly problematic in both the SARS and H1N1 events; studies have shown that people were often confused about the nature and severity of the diseases and/or about appropriate precautionary health behaviours [23, 7]. As both outbreaks were characterized by emerging and changing information, media reports tended to portray very different and sometimes alarming perspectives [24, 25]. Public attitudes on the seriousness of the disease and uptake of protective behaviours (including vaccination) were shown to vary widely between people relying on different information sources, particularly during the H1N1 outbreak [26, 27].

However, there were some notable differences in the information sources the general public could access between the two outbreaks. While Internet sources existed during SARS, there was an explosive growth of sources and types of information available through this media in the intervening years between the SARS and H1N1 outbreaks. By 2010, people were able to readily seek out information from diverse sources and perspectives, resulting in what Krimsky [28] termed the ‘democratization of knowledge’. Although this created more opportunities for acquiring information previously unavailable to the public, it did not necessarily make understanding the risk and arriving at informed decisions on health behaviours easier. Those seeking health information on the Internet were shown to experience confusion and anxiety due to the virtually unlimited amount of information, particularly if this information was different and contradictory [29]. It has been suggested that online information may have contributed to greater stress and hysteria during SARS [30]. During H1N1, Internet based sources of information were found to conflictingly provide both ‘trustworthy information’ (e.g. from public health agencies) and more biased points of view [31], and did not readily provide relevant information on prevention [32].

Greater breadth and channels of electronic information sources in recent years has further allowed people to engage in interactive online discussions through Web 2.0 platforms such as blogs, wikis and social networks. Social networking was thought to be widely used as an information source during the H1N1 outbreak and subsequent vaccination program [7]. However, as was noted for other types of Internet-based information, while some of the content available through social media was considered credible and valuable (particularly that generated through public health agencies), it was also demonstrated to be a source of misleading and bewildering information [33, 34].

In addition, access and availability of online information is differentially experienced in Canada. Despite the growing use of online media, Internet use is known to vary considerably by age group, level of education and income [35]. In 2009 only 41% of older Canadians (65 years and older) reported using the Internet at least once in the last 12 months, as opposed to 88% of those aged 35 to 54 years and 97% of those 34 years and younger. Although relatively low, this usage had increased from the mere 24% of older Canadians reporting Internet use in 2005. Internet use was also higher for Canadians with a university degree (95%) than for those with less than high school education (51%), and for those with annual incomes of greater than $50,000 (92%) than for those with annual incomes of less than $10,000 (76%).

Finally, there is evidence that new media channels do not simply replace the more traditional information channels as has been often assumed. Rather, the Internet and other forms of online interactive media are being used by information seekers to augment the use of older forms of media such as television, newspapers and radio [36]. This theory of *media or channel complementarity* [37] has been demonstrated to occur between both traditional and online media, and between interpersonal information channels (such as health care providers) and all types of mass media channels [38]. The use of the Internet as a complementary source of health information has also been shown to be more prevalent amongst younger adults than older adults [39].

## Methods

Two separate telephone surveys were conducted in the province of Alberta, Canada. The first survey was administered between October 2004 and January 2005, and was designed to examine public information seeking and perceptions of the 2003 SARS epidemic. During October 2010 a second survey was conducted to better understand public information seeking and perceptions of pandemic H1N1, and how this compared to the SARS survey results.

The responses of Albertans are considered to be generally representative of the Canadian response for both occurrences. As shown in Table 1, SARS was centred primarily in the Toronto area, and Ontario residents would thus be expected to have a unique and atypical response; Albertans were affected in a minor way similar to other provinces across Canada, and are likely more generally characteristic of the response of the majority of Canadians residing outside of Ontario. The H1N1 outbreak affected Canadians on a wider and more uniform scale, with Albertans again likely to respond similarly to the general Canadian population (Table 2).

**Table 2:** Canadian H1N1 hospitalized cases and deaths by province and territory, April 12, 2009 to April 24, 2010 (PHAC 2010).

Canadian Province/Territory	H1N1 Cases and Deaths	
	Hospitalized Cases	Deaths
British Columbia	1084	57
Alberta	1276	71
Saskatchewan	67	15
Manitoba	379	11
Ontario	1843	128
Quebec	3063	108
Nova Scotia	163	8
Newfoundland	293	7
New Brunswick	50	0
Prince Edward Island	308	18
Northwest Territories	15	3
Yukon Territory	52	1
Nunavut	85	1
**TOTAL (Canada)**	**8678**	**428**

A representative sample of Albertans was accessed through the Population Research Laboratory at the University of Alberta. The target population were those 18 years and older who, at the time of the survey, were living in Alberta and could be contacted by landline household telephones. From this population, three samples were drawn: the Edmonton metropolitan area (SARS survey n = 403; H1N1 survey n = 401), the Calgary metropolitan area (SARS survey n = 403; H1N1 survey n = 400), and the rest of the province (SARS survey n = 403; H1N1 survey n = 402), for a total of 1,209 Albertans completing the SARS survey and 1,203 Albertans completing the H1N1 survey. These sample sizes are generally representative of the provincial demographic distribution [40].

Participants were recruited using a Random-Digit Dialling (RDD) approach to ensure that residents had an equal chance to be contacted whether or not their household was listed in a telephone directory. A respondent within each household was selected on the basis of gender to ensure an equal selection of male and female participants. If contact was not made on the first call, a maximum of 10 call-back attempts were made before declaring a residential telephone number as "no contact." Ten percent of the respondents were randomly selected and re-contacted by the survey telephone supervisors for interviewing validation. The overall response rate was 47% for the SARS survey and 21% for the H1N1 survey.

Ethical approval for was obtained from the University of Alberta Faculty of Agriculture, Forestry and Home Economics Research Ethics Board (SARS survey) and the Faculties of Arts, Law and Science Research Ethics Board (H1N1 survey). As authorized by these Research Ethics Board, participants provided oral consent after being read an explanation of the survey, their rights as participants (that their participation was entirely voluntary, they did not need to answer a question, and they could terminate the interview at any time), and an assurance of confidentiality (that the information provided would be used only for the indicated purpose in conformity with the Alberta Freedom of Information and Protection of Privacy Act, the information would be stored in a secured database and used only for study purposes, the results would be analyzed only in group format and no single person would be identifiable). Participants were also provided with a name and toll free number to contact if they required further information. As is customary for telephone surveys involving no more than minimal risk and not involving procedures for which written consent is required outside the research context, the requirement for written consent was deemed to be unfeasible and waived. Oral consent was recorded as part of the overall telephone interview.

The surveys were designed with similar questions to effectively compare and contrast information seeking and information source perceptions of both outbreaks. In both the SARS and H1N1 surveys, respondents were asked how much information about SARS/H1N1 they got from nine sources: newspapers; television; radio; Internet; call lines (e.g. Health Link Alberta, a toll-free telephone service offered by Alberta Health Services that provides health advice and information); their doctor; other health professionals they know; other health professionals they don’t know; and friends and relatives who are not health professionals. An additional information source, social networking sites, was added to the H1N1 survey. Response options were based on a five point Likert scale ranging from 1 = “very little” to 5 = “a great deal”, with the option of answering “none” if the medium was not used. Respondents were also asked to rank the same information sources based on usefulness and perceived credibility of the information, again using a five point scale. During the 2004 SARS survey, respondents were asked about preferred information sources for future outbreaks.

The data was tabulated and analysed using SPSS for Windows statistical package version 18 (a product of SPSS Inc., Chicago, Illinois). Averages and frequencies were calculated for questions utilizing Likert scales, and are reported as both the percentage of people using the information source, and ‘mean scores’ reflecting the average along the 5-point scale. Results from the SARS and H1N1 surveys are reported separately to illustrate information seeking preferences during each pandemic, and to compare any changes between time periods and outbreaks. To ensure comparability of results and to account for major demographic differences between the two surveys in age and education distribution, the H1N1 data for these two demographic characteristics were weighted against the SARS data. Weighting factors range from 0.40 to 3.77. Unweighted, the mean value of the weighting factor is approximately equal to 1. A Chi-square Goodness-of-Fit test was used to compare the age, education and income respondents distributions of the two surveys against the appropriate census distributions for both Canada and the province of Alberta.

## Results

The socio-demographic characteristics of the SARS and H1N1 survey respondents are shown in Table 3. Overall, there was a shift to older, more educated and more affluent respondents in the 2010 H1N1survey as compared to the 2004 SARS survey. This is most likely due to changes in the households that could be accessed by landline telephones. The proportion of Canadian households with a residential landline decreased from 91% in 2006 (this information is not readily available for the 2004 survey year) to 67% in 2010. Concurrently, the proportion of households that use only a cell phone increased from 2.7% in 2004 to 12.8% in 2010 [41, 42]. Furthermore, the changing use of landlines is age dependent. In 2010, 80% of households comprised solely of people aged 55 and over had at least one landline, compared with 56% of households comprised of people aged 54 and under [40]. A 2008 survey showed that younger households were much more likely to use only a cell phone; 34.4% of households comprised solely of adults aged between 18 and 34 relied exclusively on cell phones, while among all other households the rate was 4.5% [43]. This change in accessibility through the survey method of random digit dialling of landline telephones is also likely the cause of the decreased response rate between the two surveys.

**Table 3:** A comparison of survey demographics (by percentage) with census data for Canada and the province of Alberta.

Socio-demographic variables		SARS2004(n = 1,209)	Canada2006(n = 31,612,897)	Alberta2006(n = 3,290,350)	H1N12010(n = 1203)	H1N12010*(n = 1203)	Canada2011^a^(n = 33,476,688)	Alberta2011^a^(n = 3,645,257)
Sex								
	Male	50.0	50.0	50.0	49.7		49.0	50.1
	Female	50.0	50.0	50.0	50.3		51.0	49.9
Age								
	18–35 years	29.7	29.7	34.6	21.0	30.0	29.5	34.5
	36–45 years	24.5	19.9	20.4	17.4	24.3	17.2	18.4
	46–60 years	29.8	27.8	26.8	34.5	30.0	28.6	27.7
	61+ years	16.0	22.6	18.2	27.1	15.7	24.7	19.4
Educational Level								
	Less than High School	8.3	23.8	23.4	8.0	8.6	n/a	n/a
	High School	33.6	25.5	26.2	20.1	33.5	n/a	n/a
	College/Post-secondary	31.9	28.1	28.9	35.9	27.0	n/a	n/a
	University Degree	26.1	22.6	21.5	36.0	31.0	n/a	n/a
Household Income								
	Less than $39,999	27.0	36.7	29.2	10.4		n/a	n/a
	$40,000—$69,999	25.3	26.6	25.5	20.1		n/a	n/a
	$70,000—$99,999	21.6	17.3	19.1	17.0		n/a	n/a
	$100,000+	26.1	19.4	26.2	52.4		n/a	n/a

* Weighted against the SARS 2004 results for age and education

^a^ Due to changes in the information collected in the Canadian census in 2011, education and income data is not available for this year.

Table 4 also compares the socio-demographics results of the SARS and H1N1 surveys (as well as the H1N1 age and education results as weighted against the SARS results) against census results for both Canada and the province of Alberta. Censuses in Canada are conducted every five years, so results for 2006 and 2011 are shown for comparative purposes (note that due to a change in the information collected in the Canadian census in 2011, education and household income data are not available for this year). For the SARS 2004 survey, the age distribution of the respondents deviates significantly from both the 2006 Canadian population (χ^2^ = 38.03, p < 0.001) and the 2006 Albertan population (χ^2^ = 25.64, p < 0.001), with a higher percentage of middle-age respondents (36 to 60 years old), and a lower percentage of older respondents (61+) (compared to both Canada and Alberta), and a lower percentage of younger respondents (18–35 years old) (compared to Alberta only). The education distribution of the SARS 2004 sample also deviates significantly from the 2006 Canadian population (χ^2^ = 166.56, p < 0.001) and of the 2006 Albertan population (χ^2^ = 159.44, p < 0.001), with a higher percentage of respondents who have at least completed high school, and a lower percentage of respondents who have less than a high school education (compared to both Canada and Alberta). Finally, the household income distribution of the SARS 2004 sample deviates significantly from the Canadian population (χ^2^ = 73.13, p < 0.001), with a higher percentage of respondents with household income of $70,000 or more, and a lower percentage of respondents with household income below $70,000. However, household income distribution of the SARS 2004 sample does not deviate significantly from the Albertan population.

**Table 4:** Mean information scores, and differences associated with age (by age group) and educational level (post-secondary versus high school or less).

	SARS								H1N1*							
Information sources	Total mean score	Mean score, x			ANOVA	Mean score, x		ANOVA	Total mean score	Mean score, x			ANOVA	Mean score, x		ANOVA
		18–35	36–55	56+	P value	Education high	Education low	P value		18–35	36–55	56+	P value	Education high	Education low	P value
Newspaper	2.69	**2.35** ^a^ ^c^	2.83	2.85	.000	2.78	2.56	.020	2.58	**2.20** ^a^ ^c^	**2.56** ^e^	3.08	.000	2.68	2.46	.032
Television	3.62	3.74	3.57	3.56	n.s.	3.59	3.65	n.s.	3.27	**3.09** ^c^	3.26	3.48	.007	3.27	3.28	n.s.
Radio	1.53	**1.35** ^b^	1.65	1.52	.020	1.64	1.39	.008	2.10	1.98	2.15	2.15	n.s.	2.42	1.45	.000
Internet	0.66	**0.82** ^c^	**0.73** ^e^	0.34	.000	0.89	0.35	.000	2.01	**2.60** ^a^ ^c^	**2.05** ^e^	1.22	.000	2.22	1.23	.000
Call Lines	0.20	0.26	0.18	0.17	n.s.	0.26	0.13	.007	0.80	**1.02** ^c^	**0.81** ^f^	0.50	.000	0.84	0.76	n.s.
Your doctor	0.32	**0.47** ^a^ ^d^	0.26	0.28	.004	0.29	0.36	n.s.	1.22	**1.50** ^a^	1.04	1.19	.001	1.30	1.11	n.s.
Known Health Professionals	0.73	**0.76** ^c^	**0.87** ^e^	0.43	.000	0.87	0.54	.000	1.45	**1.59** ^d^	1.50	1.21	.04	1.68	1.10	.000
Unknown Health Professionals	0.60	0.58	0.65	0.55	n.s.	0.65	0.54	n.s.	0.94	0.85	0.95	1.06	n.s.	1.12	0.71	.000
Friends/Relatives	1.05	**1.50** ^a^ ^c^	**0.97** ^e^	0.68	.000	1.03	1.09	n.s.	2.13	**2.74** ^a^ ^c^	**2.00** ^e^	1.64	.000	2.06	2.26	.05
Social Networking Sites		-	-	-	-	-	-	-	0.46	**0.85** ^a^ ^c^	0.33	0.22	.000	0.44	0.48	n.s.

* H1N1 data have been weighted by age and education against the SARS data to ensure comparability of results.

^**a**^ significant difference between 18–35 and 36–55 (p≤0.01)

^**b**^ marginally significant difference between 18–35 and 36–55 (p≤0.05)

^**c**^ significant difference between 18–35 and 56+ (p≤0.01)

^**d**^ marginally significant difference between 18–35 and 56+ (p≤0.05)

^**e**^ significant difference between 36–55 and 56+ (p≤0.01)

^**f**^ marginally significant between 36–55 and 56+ (p≤0.05)

The age distribution of the H1N12010 sample deviates significantly from the 2011 Canadian population (χ^2^ = 38.03, p < 0.001) and the 2011 Alberta population (χ^2^ = 120.75, p < 0.001), with a higher percentage of respondents who are 46 years old or older, and a lower percentage of young respondents (18 to 35 years old) (compared to both Canada and Alberta). The weighted age distribution of the H1N12010 sample also deviates significantly from the 2011 Canadian population (χ^2^ = 79.30, p < 0.001) and the 2011 Alberta populations (χ^2^ = 42.47, p < 0.001), with a higher percentage of middle-age respondents (36 to 60 years old), and a lower percentage of older respondents (61+) (compared to both Canada and Alberta) and a lower percentage of younger respondents (18 to 35 years old) (Alberta only).

To compensate for the shift in the Alberta population available to be surveyed using random digit dialling of landline telephones between the 2004 SARS survey and the 2010 H1N1 survey, the H1N1 survey results shown in Tables 4 and 5 have been weighted by age and education against the SARS survey results. This allows for a more direct comparison of the effects of age and education on the results obtained in the two surveys. Age was more likely to account for the variability in information source usage within both the SARS and H1N1 survey results. Not surprisingly, older respondents used traditional information sources (newspaper, television and radio) more, but used the Internet less, than younger respondents. However, interestingly, younger respondents used doctors, health professionals and friends/relatives as information sources more than older respondents. By contrast, age was not a significant factor in any of the responses on usefulness and credibility in the SARS survey. The minor age related differences noted in the responses from the H1N1 survey probably reflect the older demographic of this population.

**Table 5:** Assessed usefulness and credibility of information scores, and differences associated with age (by age group) and educational level (post-secondary versus high school or less).

		SARS								H1N1*							
		Total mean score	Mean score, x			ANOVA	Mean score, x		ANOVA	Total Mean score	Mean score, x			ANOVA	Mean score, x		ANOVA
			18–35	36–55	56+	P value	Education high	Education, low	P value		18–35	36–55	56+	P value	Education high	Education low	P value
Usefulness																	
	Newspaper	3.19	3.11	3.20	3.25	n.s.	3.23	3.12	n.s.	3.04	**2.82** ^c^	**3.01** ^e^	3.32	.000	**3.15**	**2.88**	.001
	Television	3.34	3.36	3.29	3.41	n.s.	**3.27**	**3.43**	.021	3.12	**2.98** ^d^	**3.02** ^f^	3.28	.006	3.08	3.07	n.s.
	Radio	2.66	2.54	2.66	2.83	n.s.	2.70	2.62	n.s.	2.83	**2.69** ^d^	2.84	2.98	.046	**2.90**	**2.71**	.023
	Internet	3.15	3.10	3.20	3.13	n.s.	**3.31**	**2.70**	.001	3.42	3.41	**3.52** ^f^	3.17	.048	**3.56**	**3.13**	.000
	Call Lines	2.92	2.66	2.87	3.48	n.s.	**3.17**	**2.34**	.024	3.57	3.51	**3.73** ^f^	3.11	.013	3.69	3.41	n.s.
	Your doctor	3.30	3.56	3.06	3.28	n.s.	3.33	3.25	n.s.	3.83	3.87	3.73	3.96	n.s.	**3.97**	**3.60**	.016
	Known Health Professionals	3.66	3.59	3.77	3.45	n.s.	**3.81**	**3.39**	.012	3.77	3.75	3.84	3.66	n.s.	**3.94**	**3.43**	.005
	Unknown Health Professionals	3.21	2.96	3.43	3.12	n.s.	**3.40**	**2.94**	.008	3.13	3.15	3.13	3.12	n.s.	3.20	3.02	n.s.
	Friends/Relatives	2.38	2.53	2.32	2.23	n.s.	2.39	2.37	n.s.	2.65	**2.89** ^c^	2.48	2.64	.000	2.60	2.74	n.s.
	Social Networking Sites	-	-	-	-	-	-	-	-	2.23	2.33	2.17	2.04	n.s.	2.13	2.37	n.s.
Credibility																	
	Newspaper	3.30	3.36	3.26	3.32	n.s.	3.32	3.27	n.s.	3.09	3.05	3.05	3.19	n.s.	**3.16**	**2.98**	.026
	Television	3.37	3.32	3.33	3.50	n.s.	**3.27**	**3.51**	.000	3.10	3.03	3.07	3.21	n.s.	3.12	3.04	n.s.
	Radio	3.07	2.98	3.02	3.28	n.s.	2.98	3.14	n.s.	2.91	2.80^d^	2.90	3.08	.046	2.98	2.80	.034
	Internet	3.25	3.21	3.27	3.32	n.s.	**3.39**	**2.87**	.004	3.34	3.33	3.42	3.16	n.s.	**3.44**	**3.14**	.003
	Call Lines	3.29	3.00	3.24	3.90	n.s.	**3.58**	**2.61**	.007	3.75	3.67	3.93	3.45	n.s.	3.85	3.61	n.s.
	Your doctor	3.78	3.98	3.57	3.80	n.s.	3.80	3.78	n.s.	4.03	4.05	3.96	4.12	n.s.	**4.20**	**3.76**	.000
	Known Health Professionals	4.03	3.91	4.12	4.03	n.s.	**4.19**	**3.76**	.003	3.91	3.89	3.96	3.84	n.s.	**4.10**	**3.55**	.000
	Unknown Health Professionals	3.51	3.42	3.58	3.48	n.s.	**3.75**	**3.16**	.000	3.26	3.32	3.26	3.21	n.s.	**3.39**	**3.06**	.028
	Friends/Relatives	2.50	2.53	2.44	2.56	n.s.	2.55	2.44	n.s.	2.61	2.75^b^	2.50	2.60	.038	2.60	2.63	n.s.
	Social Networking Sites	-	-	-	-	-	-	-	-	2.06	2.13	1.96	2.08	n.s.	2.00	2.15	n.s.

* H1N1 data have been weighted by age and education against the SARS data to ensure comparability of results.

^**a**^ significant difference between 18–35 and 36–55 (p≤0.01)

^**b**^ marginally significant difference between 18–35 and 36–55 (p≤0.05)

^**c**^ significant difference between 18–35 and 56+ (p≤0.01)

^**d**^ marginally significant difference between 18–35 and 56+ (p≤0.05)

^**e**^ significant difference between 36–55 and 56+ (p≤0.01)

^**f**^ marginally significant between 36–55 and 56+ (p≤0.05)

Instead, education was more likely to account for the variability in the identified usefulness and perceived credibility of each information source within the two surveys. Educational differences were particularly notable for the Internet and known health professionals, which were deemed to be both more useful and credible by those with higher education (post-secondary, including any education following high school) in both surveys. Education effects were also observed between the two surveys. Television was seen as less useful and less credible by those with higher education in the SARS survey, but not in the H1N1 survey. Educational differences in the usefulness and credibility of call lines, such as Health Link, seen during the SARS survey were not evident in the H1N1 survey. Doctors were considered to be more useful and credible as information sources by those with higher education only in the H1N1 survey.

**Fig 1:**
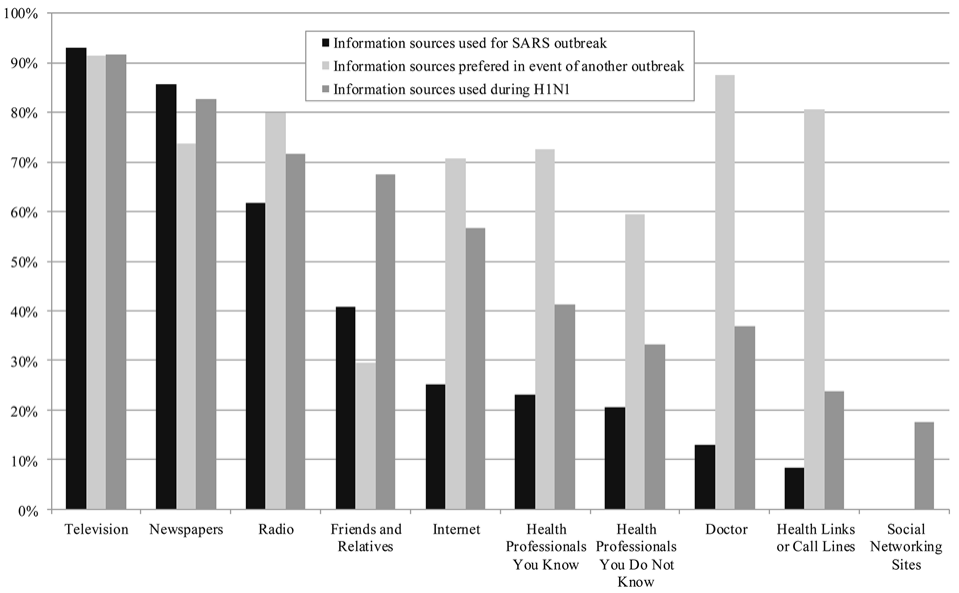
Information sources used during SARS & H1N1, and those indicated as preferred sources in a future outbreak during SARS.

The information sources used by survey participants during SARS and H1N1 are shown in both Fig 1 and Table 4. Fig 1 illustrates the total percentage of respondents reporting using a particular information source. It also shows the information sources respondents from the SARS survey stated they would prefer to access in the event of a future outbreak. Table 4 provides the mean score for information use, thus indicating the degree to which each source was utilized. Traditional mass media sources (newspaper, television and radio) remained the most commonly used sources by both the SARS and H1N1 survey respondents. As anticipated, there was an increase in Internet as an information source, from 25% during SARS to 56% during H1N1. Surprisingly, the number of people using social media as an information source during H1N1was not as large as anticipated (only 17%). This may again be an artefact of the older population represented in this survey.

Two rather interesting patterns emerged between the two survey periods in terms of accessing more interpersonal information sources. Respondents accessed friends and family as information sources more during H1N1 than SARS (27% increase). They also accessed doctors (24% increase), known health professionals (18% increase), call lines (16% increase), and other health professionals not personally known by the respondents (13% increase) more during H1N1. Notably, these changes in information sources correspond to the information sources identified by the SARS survey respondents as those they would prefer to access during a future outbreak.

However, when this same set of sources are examined for their usefulness and credibility, a slightly different pattern emerges (Table 5). Friends and relatives ranked the lowest in terms of mean scores for usefulness and credibility for both the SARS and H1N1 surveys (together with social networking sites in the H1N1 survey). In both surveys, known health professionals and doctors were deemed the most useful and credible. Television was also ranked fairly high as being both useful and credible (and in fact was deemed to be slightly more useful than doctors in the SARS survey), together with health professionals not known to the respondents. Of particular interest are the differences between perceived usefulness and credibility for doctors and call lines, both of which were considered to be more credible than useful.

**Fig 2:**
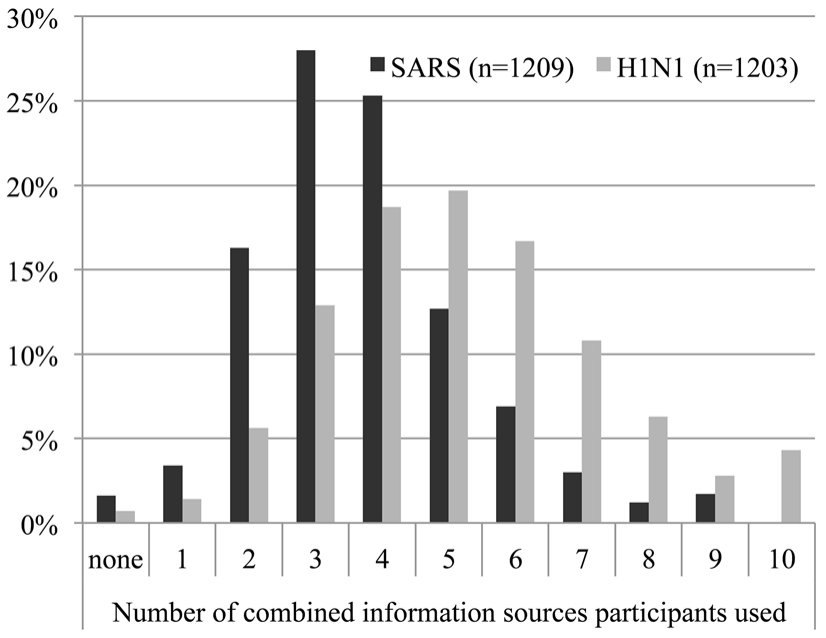
Number of combined information sources used during SARS and H1N1.

Finally, there is a considerable increase in the number of combined information sources accessed by respondents from SARS to H1N1. The number of respondents reporting that they used five or more information sources during H1N1 increased by almost 60% compared to the SARS survey (Fig 2). The same pattern holds for combined information sources that respondents indicated that they used ‘often’ or ‘a great deal’. Respondent use of single information sources dropped (39% during SARS to 22% during H1N1) and the use of three or more combined sources increased (14% during SARS to 41% during H1N1).

## Discussion and Conclusions

Public health risk communication in the 21^st^ century is continuously changing. It is critical for public health agencies and practitioners to better understand the shifting landscape of communication mediums and the public’s current use and preference for information sources. Four major findings from this research have particular relevance for public health risk communication during both future infectious disease outbreaks and for other health issues.

First, public health researchers and practitioners need to reconsider the representativeness of information obtained using telephone surveys. Random digit dialled telephone surveys of landline telephones are still commonly used to measure public risk perceptions and behavioural attitudes during an infectious disease outbreak because they are relatively rapid and easy to implement, and can thus inform policy decisions and shape health risk communications in a timely manner [44]. However, this comparative research demonstrated that the changing demographic accessed using this type of survey has significant implications for the interpretation of the information (in terms of population representativeness) and for assessing shifts in attitudes and behaviours over time. In the future, public health agencies and researchers using this data collection mechanism will need to make more concerted efforts to access a younger age demographic that may rely more on cell phones and electronic communications [45].

Second, there was not the expected magnitude of increase in the use of Internet and social media, with the majority of respondents still relying primarily on traditional media information sources. This finding is particularly relevant given the realities of our aging population and their increasing needs for health information [46]. This pattern signals that the growing emphasis on providing official public health risk communication and information through Internet and social media sources, while important, should not replace the use of more traditional information channels. In addition, different online information seeking patterns for older adults means that material that is provided through these channels needs to be tailored for different types of audiences [47, 48]. Although it is recognized that Internet usage has changed since the 2010 H1N1 survey, national surveys show increases are fairly modest. The 2012 Canadian Internet Use Survey [49] found that the percentage of Canadian homes with access to Internet only increased by 4% from 2010 (from 79% to 83%). Furthermore, the majority (61%) of those without home Internet access reported they had no need for or interest in it, thus continuing to validate the need for multiple sources and channels of health information.

Third, there is a disconnect between the information sources people use and find useful, and those they consider most credible. Although respondents most frequently used traditional mass media and friends/relatives as information sources, they deemed known health professionals, doctors and call lines (such as Health Link) the most credible sources. Furthermore, respondents from both surveys found that doctors and call lines were more credible than useful as information sources. There is an obvious need to better understand the barriers people have in accessing and using these trusted sources of information during an infectious disease outbreak. This finding also highlights the need to improve communication and knowledge transfer to health care providers [7, 50, 51], and to provide them with opportunities to be more prominently involved as spokespeople during infectious disease outbreaks.

Finally, and perhaps most importantly, public health agencies need to be aware that people are taking advantage of the unprecedented number of potential health information channels by increasingly using multiple sources to augment and complement their information searches. This further reinforces the need to use multiple channels (both traditional and online) for health risk communications. It also means that agencies and public health professionals need to recognize that other sources may have conflicting or confusing information that is contrary to primary public health recommendations (e.g. vaccine safety), and that they have an obligation to address these issues directly in public health risk communication messages. ‘More is better’ only if the additional information sources can contribute to better informed decision-making; otherwise use of multiple sources can create confusion about agency credibility and appropriate health risk behaviours that may cause more harm than good.

## Limitations

The limitations to this study are related to the telephone survey method and the changing demographic that can be accessed using this type of data collection. As shown through the comparison to census data, the sample accessed through these surveys is significantly different than both the Canadian and Albertan census populations, with higher proportions of older, more educated and higher income respondents. The reduction in the use of landlines between 2004 and 2010, particularly among older people, further skewed the respondent demographics in the second (H1N1) survey towards an older and more educated group. As this segment of the population is also known to rely less on online sources of information, this method limitation may also have influenced the results of this study.
